# Clustering mechanism of ethanol-water mixtures investigated with photothermal microfluidic cantilever deflection spectroscopy

**DOI:** 10.1038/srep23966

**Published:** 2016-04-05

**Authors:** M. S. Ghoraishi, J. E. Hawk, Arindam Phani, M. F. Khan, T. Thundat

**Affiliations:** 1 Department of Chemical and Material Engineering, University of Alberta, Edmonton, Canada

## Abstract

The infrared-active (IR) vibrational mode of ethanol (EtOH) associated with the asymmetrical stretching of the C-C-O bond in pico-liter volumes of EtOH-water binary mixtures is calorimetrically measured using photothermal microfluidic cantilever deflection spectroscopy (PMCDS). IR absorption by the confined liquid results in wavelength dependent cantilever deflections, thus providing a complementary response to IR absorption revealing a complex dipole moment dependence on mixture concentration. Solvent-induced blue shifts of the C-C-O asymmetric vibrational stretch for both anti and gauche conformers of EtOH were precisely monitored for EtOH concentrations ranging from 20–100% w/w. Variations in IR absorption peak maxima show an inverse dependence on induced EtOH dipole moment (*μ*) and is attributed to the complex clustering mechanism of EtOH-water mixtures.

Aqueous mixtures of alcohol have been under investigation among biologists, physicists, chemists and engineers due to its relevance in many areas such as the pharmaceutical industry, biofuels, and protein aggregation^1^,^2^,^3^,^4^,^5^,^6^,^7^. Alcohol-water mixtures overcome limitations of the poor solubility of pure hydrophobics in water and thus, are of particular importance in the study of protein folding and stability as well as hydrophobic solvation^8^,^9^,^10^,^11^,^12^, where solute-solute, solvent-solvent and solvent-solute interactions play an important role. As a result, short chain alcohols have been used as a simple model that may facilitate understanding of more complex aqueous solutions such as biomolecules^9^,^10^,^11^,^12^,^13^.

Of the short chained alcohols, EtOH is one of the most popular and has been studied with a variety of computational and experimental techniques^14^,^15^,^16^,^17^,^18^ to include Raman (Burikov *et al.*)^19^ and NMR and FT-IR spectroscopy (Mizuno *et al.*)^20^. EtOH consists of an equilibrium mixture of two conformers, anti and gauche, each of which exhibits an associated absorption band in IR^21^,^22^. C-O stretch in alcohol,more appropriately considered as C-C-O asymmetric stretch in EtOH, displays two distinguishable peaks in the 1000–1100 cm^−1^ region with approximately 40 cm^−1^ wavenumber difference, each related to the anti and gauche conformers, respectively^22^,^23^. The band around 1045 cm^−1^, due to the more favorable anti conformer, has the more intense peak among the two bands and is often the peak referred to in studies^22^. Here we refer to C-O stretch as C-C-O asymmetric stretch while investigating the absorption of both the anti and gauche conformer^23^.

In this letter we report on shifts in the vibrational energy peaks of EtOH water mixture experimentally observed using micromechanical calorimetric spectroscopy using bimaterial microfluidic cantilevers (BMC). The experimentally measured shifts in the wavenumber at IR absorption peak maxima show (1/*μ*) dependence. From first principles we show that IR absorption wavenumber is proportional to (1/*μ*), which anticipates a nonlinear relation between IR absorption wavenumber and concentration of EtOH as observed.

We fabricated microfluidic channels on bimaterial cantilevers (BMC) capable of holding 115 ± 1% pL of liquid. We have used a quantum cascade laser (QCL) as a light source which solves the low spectral power density of IR spectroscopy. By incorporating QCL and BMC, we were able to obtain nonlinear blue shift of C-C-O vibration as a result of nonlinear changes in dipole moment of EtOH as predicted by molecular dynamic simulation^24^.

Photothermal cantilever deflection spectroscopy is based on measuring extremely small changes in thermal energy due to absorption of infrared energy by molecules. When a molecule is illuminated by IR radiation, it absorbs the radiation that matches the vibrational energy of the bond. This vibrational energy will be lost as heat during the relaxation process. The heat generated during the relaxation process is monitored as bending of a bimaterial cantilever. Photothermal deflection of the microchannel cantilever as a function of wavenumber provides the IR spectrum of the samples^25^,^26^.

Using Hooke’s law, the vibrational frequency of a chemical bond, where atoms and the connecting bond are modeled as a simple harmonic oscillator, can be expressed as,


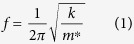


where, *f* is frequency of oscillation, *k* is force constant and m* is reduced mass. Frequency of oscillation is related to wavenumber by *v* = *f*/*C*, where *C* is wave velocity. In the presence of an electric field, E, the equilibrium position of a molecule carrying charge *dq* will be shifted by *dx* = *Edq*/*k* which results in the induced dipole moment *μ* = *Edq*^2^/*k*. As a result of change in the charge separation on the molecule, the force constant associated with bonds in the molecule will be different. Therefore, IR light with slightly different energy (frequencies) will be absorbed, matching to the bond stiffness change. Substituting k obtained from the induced dipole relation into Equation (1), we can relate changes in the IR absorption wavenumber with the internal electric field in the binary mixture as a result of polar media surrounding the analyte.


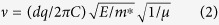


In addition, induction effects and Keesom forces in binary mixtures of water-EtOH is expected to lead to varying molecular dipole moments of both water and EtOH as concentration changes.

## Results

**Figure 1:**
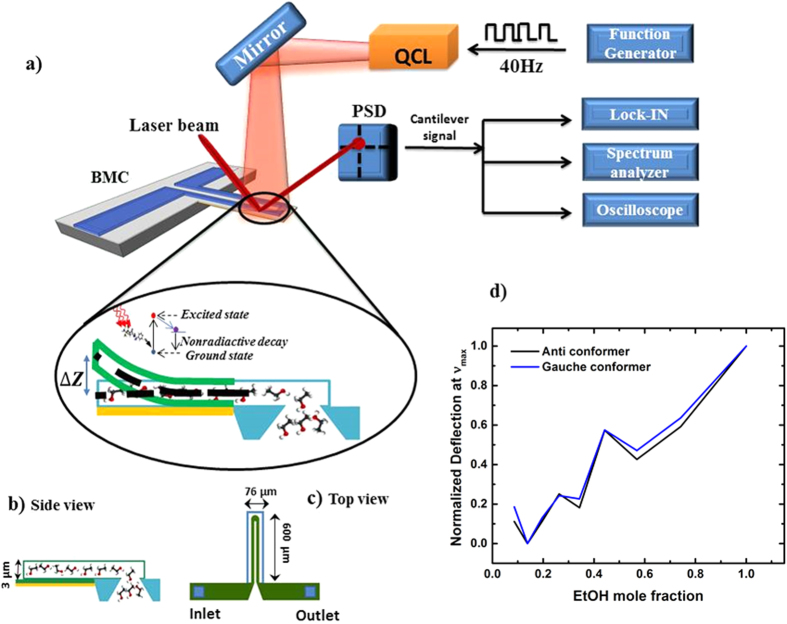
(**a**) Experimental set-up to collect IR spectrum of EtOH-water mixtures, (**b**) cross-section of the microchannel cantilever, (**c**) top view of the microchannel cantilever and (**d**) variation in bimaterial microfluidic cantilevers (BMC) deflection at absorption maxima (*v*_max_) for each concentration.

**Figure 2:**
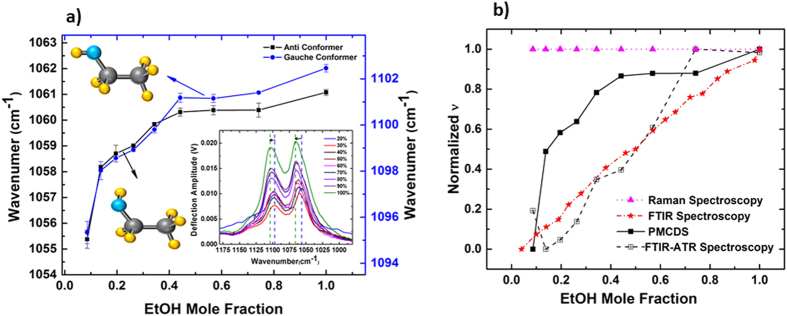
(**a**) effect of concentration on IR peak maxima (*v*) for EtOH anti and gauche conformers. Concentration of EtOH changes from 20 wt% to 100 wt%. Inset graph shows IR spectrum of binary mixtures collected using PMCDS method where purple dash line are fixed at peak maxim for EtOH 20 wt% and olive dash lines are fixed at EtOH 100 wt%. Error bar refers to the standard deviation of the peak maxima from mean peak maxima at each concentration. (**b**) Normalized peak maxima for anti conformer as a function of concentration using Raman, FTIR, FTIR-ATR and photothermal spectroscopy. (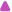) shows reported results of Burikov *et al.* using Raman spectroscopy, (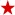) shows results of Mizuno *et al.* using FTIR spectroscopy, (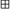) and (■) show our experimental results using FTIR-ATR spectroscopy and PMCDS respectively.

The IR spectra of binary mixtures of EtOH-water as concentration changes from 20–100 wt% were experimentally collected using a BMC. The experimental setup, device schematic and normalized deflection intensity at different concentrations are shown in Fig. 1. Each concentration was tested three times. After deconvolution of each spectrum, using a Voigt function, the average peak maxima for both anti and gauche conformers were plotted as a function of concentration (Fig. 2a). Deconvolution of each spectrum is provided in supplementary material, Figs S1–S3. To compare our results with the reported results of FTIR and Raman studies, the peak maxima for the anti conformer was normalized and plotted as a function of concentration (Fig. 2b) alongside the normalized reported results for C-C-O asymmetrical stretch at approximately the same wavenumber using FTIR and Raman spectroscopy. While our results show a nonlinear relation between absorption intensity as concentration increase, results of Burikov *et al.* and Mizuno *et al.* show no change or linear change, respectively.

**Figure 3:**
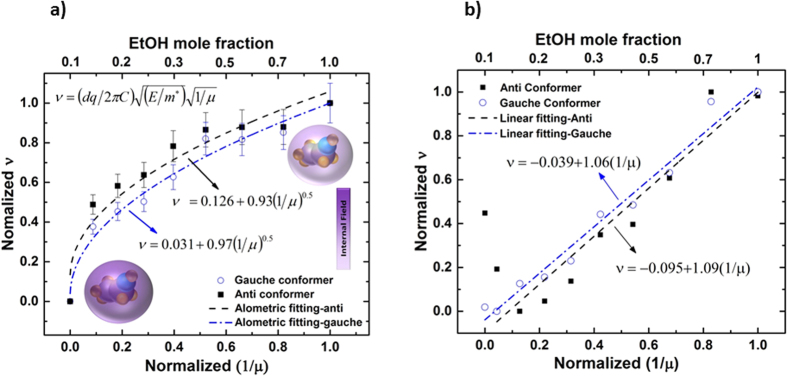
*v* (peak maxima) dependence on (1/*μ*) of EtOH, as concentration of EtOH changes from 20 wt% to 100 wt%. The dipole moments correspond to mixture concentrations in experiments as derivable from ref. 24 presented in a normalized scale. (**a**) peak maxima from PMCDS experiments. Normalized peak maxima vs normalized (1/*μ*) follows a power law relation as shown, with least square of 92% for anti and 97% for gauche conformer. (**b**) Peak maxima for this figure are related to ATR-FTIR spectrum. Normalized peak maxima relation with normalized (1/*μ*) follows the linear relation shown, with least square of 87% for anti and 97% for gauche conformer. The 20 wt% data point was excluded for linear fitting of the anti conformer due to high enthalpies of mixing for this concentration.

IR absorption peak maxima wavenumber for both anti and gauche conformer was normalized for each concentration and plotted vs scaled dipole moment of EtOH correlated with the same concentration (Fig. 3a). The relationship between IR absorption wavenumber, *v*, for both anti and gauche conformers of EtOH, and inverse dipole moment, 1/*μ*, of EtOH at different concentrations follows a power law dependence agreeing with the empirical equation (2), with a least squares agreement of 92% for anti and 97% for gauche conformers^21^. The slope of *v* and 1/*μ* dependence decreases as EtOH concentration increases. As can be seen from equation (2), the slope should be proportional to the electric field perceived by EtOH molecules, and their effective mass, as a function of concentration. Such power law dependence indicates an effective change in (*E*/m*) due to changes in clustering present in the mixture, which results from changes in the concentration. This result is consistent with a decrease in dipole moment of EtOH in the binary mixture as EtOH concentration increases^24^.

For comparison, employing the attenuated total reflection technique, spectrums of the EtOH-water mixtures at different concentrations were collected. ATR-FTIR spectra of the binary mixtures are shown in supplementary material, Fig. S4. To investigate the relationship between absorption peak maxima and dipole moment, *v* vs 1/*μ* was plotted for both anti and gauche conformers. As it is shown, *v* is linearly dependant on 1/*μ* with least square of 87% for anti and 97% for gauche conformer (Fig. 3b). This result shows that even though ATR-FTIR spectroscopy is a powerful technique and requires a smaller volume of sample compared to regular FTIR spectroscopy, it may not be optimal for studying intermolecular interactions of volatile samples. This could be as a result of having an open system which increases the chance of evaporation of alcohol and consequent changes in the concentration. For concentrations less than 30 wt%, the anti conformer peak maxima wavenumber increases considerably. This is possibly as a result of higher enthalpy of mixing for concentrations less than 30 wt% since the dipole moment of EtOH can be effected by increase in temperature^27^.

In order to study IR absorption intensity at a fixed wavenumber as a function of concentration, cantilever deflection was plotted at different concentrations. The effect of concentration on absorbance is routinely related by Beer-Lambert’s law, *A* = *εlc*, where absorbance, *A*, is related to concentration, *c*, extinction length, *l*, and absorptivity, *ε*, which depends on (∂*μ*/∂*x*)^2 ^23. Changes in the transition moment (∂*μ*/∂*x*), as perceived by an IR wave at different concentrations for clustering effects, would cause a variance in wave-matter interaction with molecules and thus can change the overall absorbance magnitude. Since the laser light’s electric field has the same polarization for all samples in our experiments, the orientation of the EtOH as a result of clustering is the controlling factor, influencing the extent of change in vibrational transition moment angles (VTMAs).

**Figure 4:**
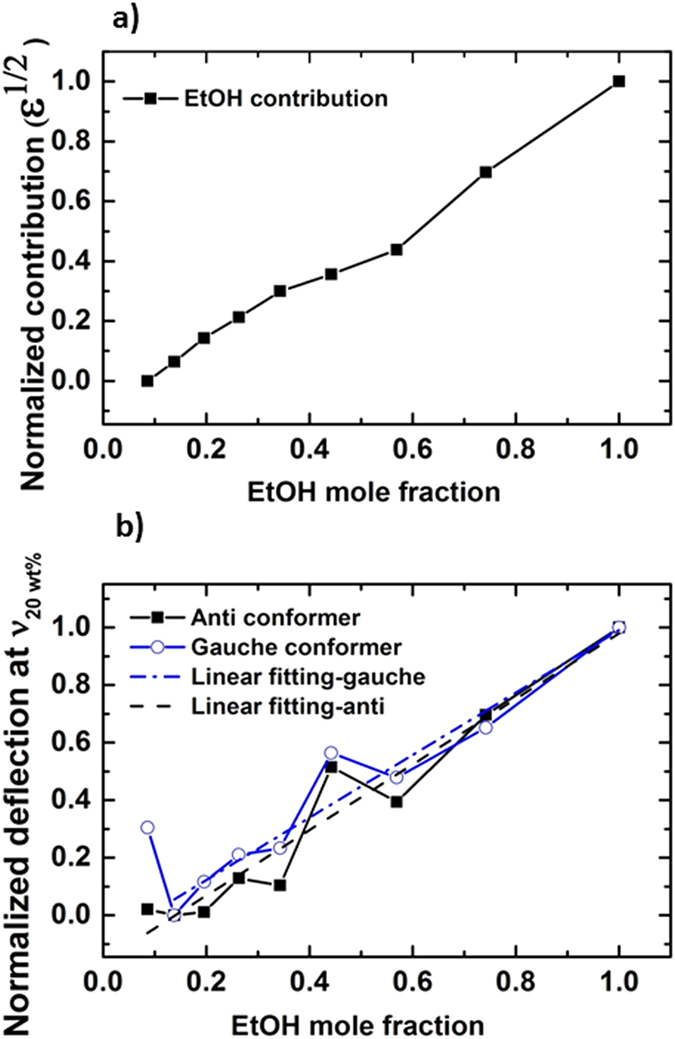
IR absorbance intensity relation with concentration, (**a**) normalized variation in 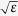 of EtOH in the binary mixture as concentration changes (unprocessed data are from ref. 24), (**b**) change in cantilever deflection for both anti and gauche conformer at different concentrations while wavenumber is fixed at peak maxima of 20 wt% (*v*_20−*wt*%_). Linear fits of both anti and gauche conformer show least square of 93%. (Anti: *d* = −0.16 + 1.14*X*_*EtOH*_ Gauche: *d* = −0.1 + 1.08*X*_*EtOH*_, d represent the normalized deflection of the cantilever at *v*_20−*wt*%_ and *X*_*EtOH*_ shows EtOH mole fraction).

To investigate any possible contribution of the dipole moment of EtOH on cantilever deflection we used published data from ref. 24, where contributions of EtOH component to dielectric constant of the mixture was computed from fluctuations of dipole moment of the simulation box


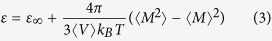


where M is total dipole moment of the box and *ε*_∞_ (high frequency contribution) is 1.69^24^. Our results show EtOH contribution to cantilever deflection intensity changes as a function of concentration, which agrees with the contribution dependence of fluctuations of *μ* within the simulation box for clustering effect at different concentrations. Fluctuation of *μ* is presented in terms of 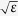, *ε* being dielectric constant, (Fig. 4)^24^. Even though overall change in the deflection as a function of concentration shows a linear trend, consistent with Beer-Lambert’s law, there are anomalous points at 20 wt% and 70 wt%. One can conclude that, since absorbance intensity does not increase linearly with concentration for all concentrations, changes in the transition dipole moment are not linearly increasing with concentration, revealing clustering as reported by neutron diffraction measurement and molecular dynamic (MD) models performed with approximately 10^8^ molecules, whereas our experiments are done with approximately 10^15^ molecules^1^,^24^. At X_EtOH_ = 0.7 most of the water forms small clusters and only a small fraction of the water compound is monomeric^24^. In contrast, X_EtOH_ slightly less than 0.5 has been reported as the critical percolation point of water network^2^,^24^. This could explain why the IR absorption intensity at X_EtOH_ = 0.44 is higher than X_EtOH_ = 0.57, which cannot be explained by Beer-Lambert’s law.

At very low concentrations, water molecules are reported to be densely packed around the hydrophobic tail of alcohol resulting in a compressive effect from water which causes the minimum in partial molar volume at X_EtOH_ = 0.07 28. At this concentration all water are in the hydration shell of EtOH^28^. Perceptible higher amplitude of deflection at 20 wt% for gauche conformer could be as a result of such existence of waters in the hydration shell of EtOH which possibly make the presence of gauche conformer more favourable than anti in the mixture.

Although other experimental techniques could not directly measure the distribution of molecular dipole moment in the condensed phase, such changes could possibly be reflected in the response of our system. Local (electro)chemical environment can not only change the average value of electrostatic properties, such as dipole moment of molecules, but also the distributions of molecular dipole moment. Our results shown in supplementary, Figs S1–S3, reveal that the spectrum of the binary mixture at 20% w/w, where solution is dominated by water molecules, has a lower Q-factor than those with higher concentrations of EtOH. The lower Q-factor corresponds to higher dissipation or relaxation losses and is in agreement with reported broader dipole moment distribution at X_EtOH_ = 0.1, comparing to X_EtOH_ = 0.5 and X_EtOH_ = 0.9 due to stronger polarization of the EtOH by the bulk water-like environment^29^.

## Conclusion

We have investigated the effect of solvent-solute interactions on the fundamental C-C-O vibrations of EtOH by using micromechanical calorimetric spectroscopy to collect IR spectrum of EtOH-water mixtures. This technique offers a means to study and understand dipole dependence on molecular vibrations in confined picoliter volumes of mixtures, previously unexplored due to limitations of volume levels in other analytical techniques. Our results reveal power law dependence of the IR absorption peak maxima on induced dipole moment of EtOH in the mixture. Also, non-linear contributions of EtOH in the IR absorption intensity at fixed wavenumber show the important effect of clustering in the vibrational transition moment, which cannot be explained by Beer-Lambert’s law. This technique allows correlation to MD simulations with experimental results where the total number of molecules in question are comparable, as well as liquid matter sensing and product verification. Further investigations may elucidate such complex inter and intra-molecular mechanisms in wave-matter interactions.

## Methods

### Measurement and experimental setup

IR spectroscopy of the ethanol-water mixtures were collected using the photothermal deflection of the microfluidic channel cantilever, where thermal heating is induced by photon absorption. Absolute ethanol was purchased from Sigma Aldrich with concentration higher than or equal to 99.8%. Water was milli-Q water (purified using Milli-Q Advantage A10). The ethanol concentration in the prepared solutions was changed from 20% to pure ethanol in 10% w/w increments. A silicon nitride cantilever with a microfluidic channel constructed on the top, and 250 nm of Au coated on the opposite side was used in the current experiments (Fig. 1). Helium Ion Microscope (HiM) image of microfluidic cantilever top view, and the microchannel constructed on cantilever is provided in supplementary material, Fig. S5. Thermally induced bending of the microchannel cantilever is recorded by tracking the position of a laser beam reflected off of the cantilever onto a position sensitive diode (PSD) whose output voltage is proportional to the cantilever’s bending.

The output signal from the PSD is fed to a lock-in amplifier (SRS 850 Stanford Systems) and spectrum analyser (SRS 760 Stanford Systems) to monitor the frequency and amplitude of the cantilever vibration as various concentrations of EtOH-water mixtures are loaded into the channel. The photothermal spectrum of each mixture is obtained by illuminating the cantilever with monochromatic infrared radiation using a quantum cascade laser (QCL) (Daylight Solutions) which is pulsed at 40 Hz using an SRS DS345 function generator. The IR beam, with wavelengths varying from 8.3 μm to10.4 μm, is focused onto the cantilever (Fig. 1). In addition, the photothermal spectrum (provided in Supplementary material Fig. S6) of the SiN cantilever filled with water is collected and used for reference correction. Samples were positioned into the lever by applying negative pressure at the outlet of the BMC chip.

A Thermo Scientific Nicolet, Nexus 670 system with Smart Performer ZnSe window was used to collect ATR-FTIR spectrums of the binary mixture. Even though 40 μl of liquid was enough to cover the ZnSe window while collecting IR spectrums, 100 μl of the mixture was placed on the ZnSe window in order to reduce the effect of EtOH evaporation in concentration during measurement. Each measurement takes about 55 Seconds. The ZnSe window was washed with EtOH and milli-Q water and then air dried after each measurement.

### Fabrication

The microcantilevers were fabricated on a silicon substrate using both surface and bulk micromachining methods. The fabrication process of a microchannel cantilever begins with deposition of 0.5 *μm* of silicon rich silicon nitride (SRN) using standard low pressure chemical vapor deposition (LPCVD) technique followed by photolithography and reactive ion etching (RIE) to define inlet/outlet square holes. In order to define the microfluidic channel, a 3 *μm* sacrificial layer of polysilicon is deposited on top of the SiN layer. The thickness of sacrificial polysilicon defines the height of the microchannel located on top of the cantilever base; this layer will be etched later. This is followed by photolithography and RIE to define the microchannel structure. A second layer of SRN is deposited with a thickness of 0.5 *μm*. Then, the double layer of SRN is etched using RIE to define the underlying cantilever. On the backside of the wafer, SRN and polysilicon are etched to make a hole under the cantilever which allows for deposition of Au on the backside of the cantilever after it is released. Finally, the wafer is placed in a 28% KOH bath at 80 °C in order to anisotropically etch the silicon from the bottom side of the wafer. After etching through the wafer, KOH reaches the sacrificial layer on the top side of the wafer and begins etching it. KOH etching process takes more than 20 hours to be completed.

## Additional Information

**How to cite this article**: Ghoraishi, M. S. *et al.* Clustering mechanism of ethanol-water mixtures investigated with photothermal microfluidic cantilever deflection spectroscopy. *Sci. Rep.* **6**, 23966; doi: 10.1038/srep23966 (2016).

## Supplementary Material

Supplementary Information
