# Supplementary material for: Clustering mechanism of ethanol-water mixtures investigated with photothermal microfluidic cantilever deflection spectroscopy
Source: Sci Rep. 2016 Apr 5;6:23966. doi: 10.1038/srep23966 (PMC4820714; doi:10.1038/srep23966)
Supplement: Supplementary Information [file srep23966-s1.pdf]

Supplementary information:

Clustering mechanism of ethanol-water mixture investigated with Photothermal microfluidic cantilever deflection spectroscopy

M. S. Ghoraishi*, J. E. Hawk, Arindam. Phani, M. F. Khan, T. Thundat*

Section A) IR spectrum obtained using photothermal microfluidic cantilever deflection spectroscopy (PMCDS) when concentration of EtOH changes from 20 wt% to 100 wt% is deconvoluted using Voigt function. Each concentration was tested three times and here we show only first run of experiments. Following equation shows Voigt function used in this work.

$$y = y_0 + A\left[\gamma \frac{2}{\pi} \frac{w}{4(x-x_c)^2 + w^2} + (1-\gamma)\frac{\sqrt{4\ln 2}}{w\sqrt{\pi}} e^{\frac{-4\ln 2}{w^2}(x-x_c)^2}\right]$$

Where, y is profile intensity, y_0 is offset, x_c is center wavenumber, A is area, w is full width half maxima (FWHM) and γ is profile shape factor.

For comparison, spectrum of 100 wt% EtOH obtained using ATR-FTIR is shown in Figure S3.

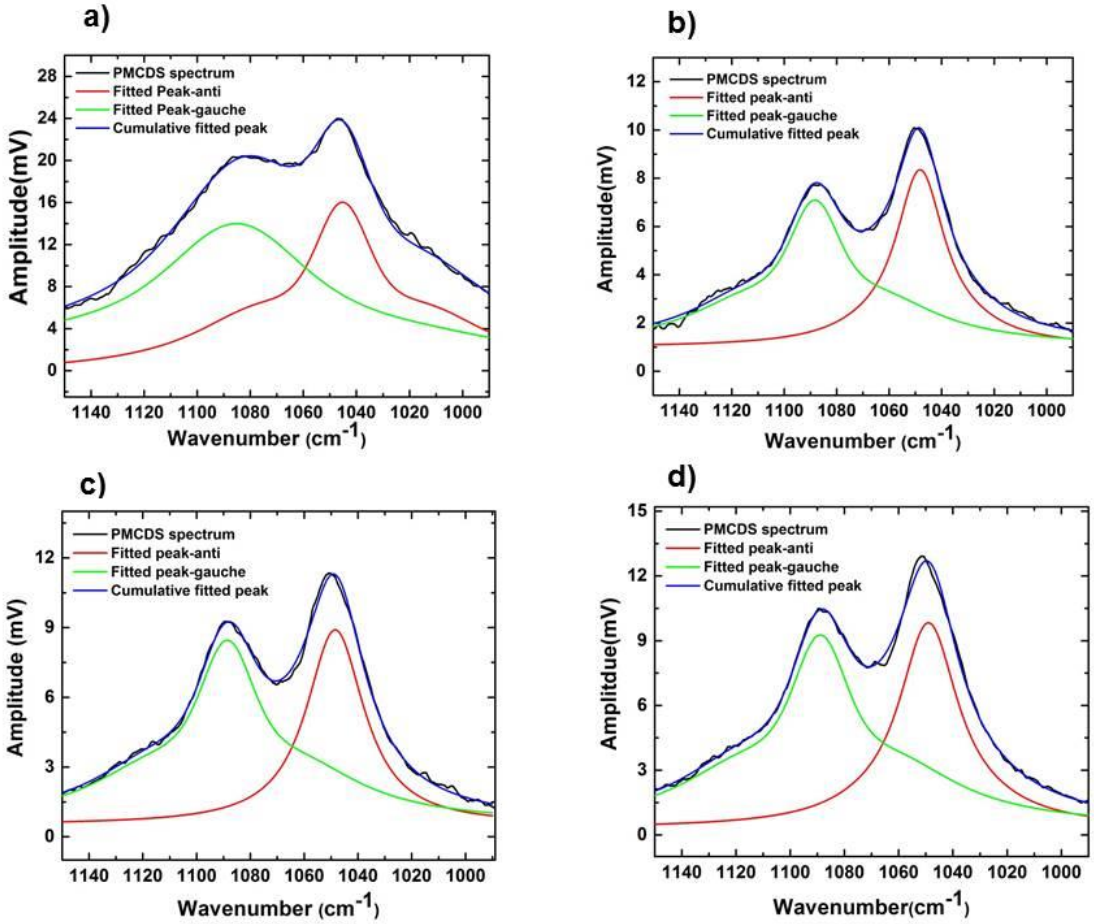

Supplementary Figure S1: IR spectrum obtained by PMCDS and deconvolutions of spectrum using Voigt function for the following concentrations of EtOH a) 20 wt% b) 30wt% c) 40wt% and d) 50wt%.

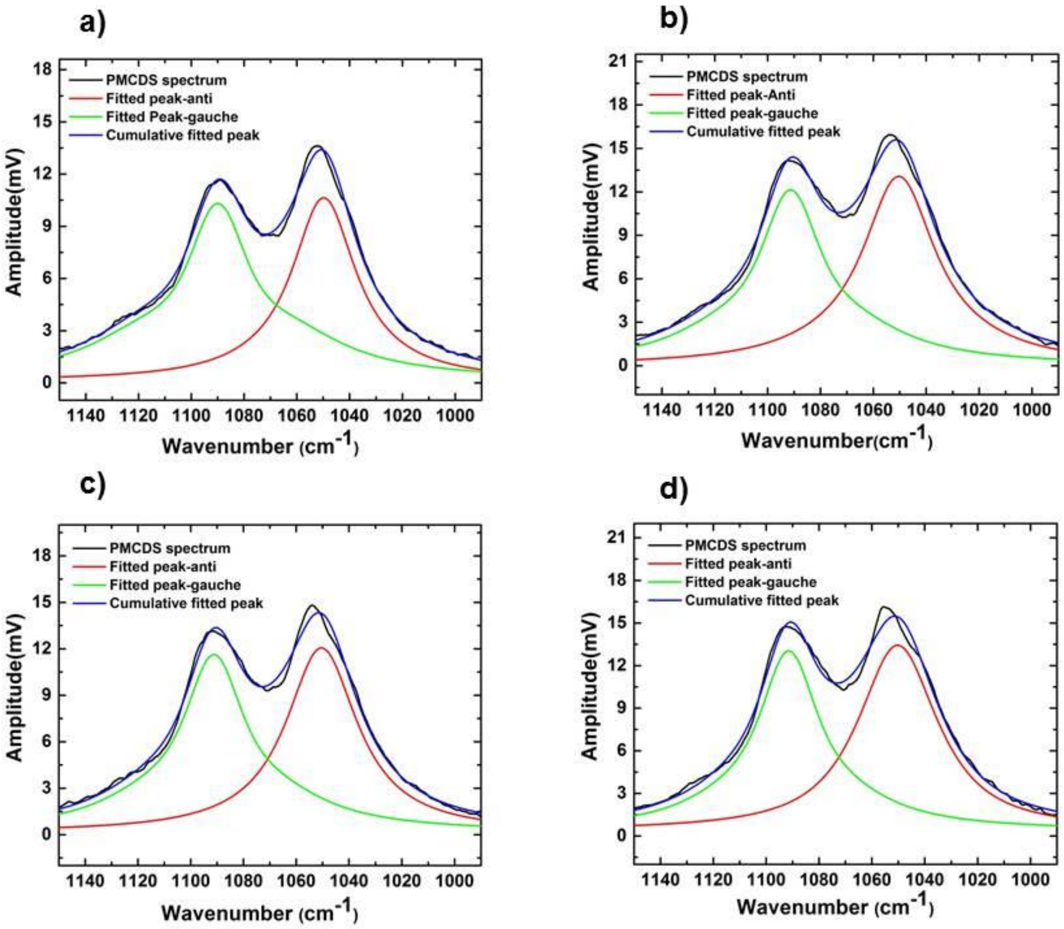

Supplementary Figure S2: IR spectrum obtain by PMCDS and deconvolutions of spectrum using Voigt function for the following concentrations of EtOH a) 60wt% b) 70wt% c) 80wt% and d) 90wt%.

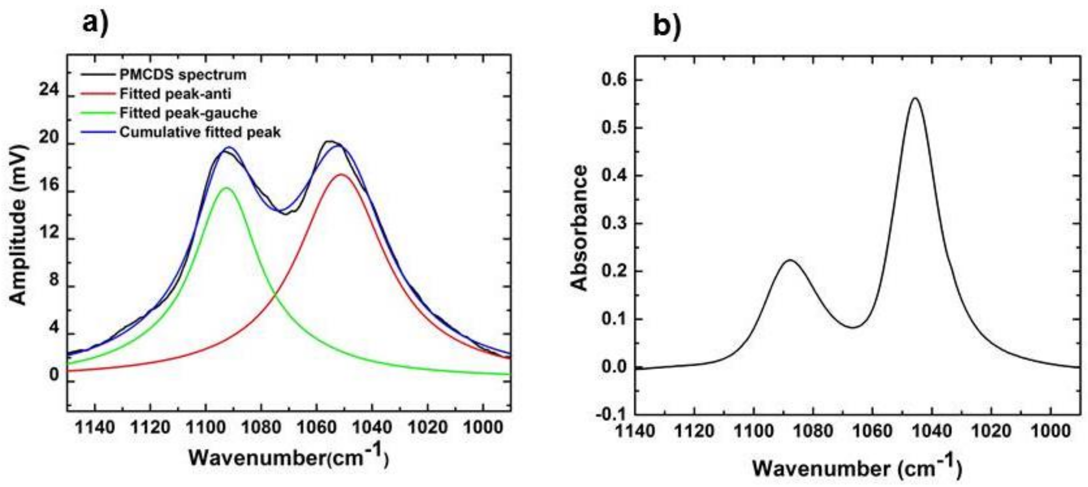

Supplementary Figure S3: IR spectrum obtained employing a) PMCDS and b) ATR-FTIR spectrum for 100 wt% EtOH

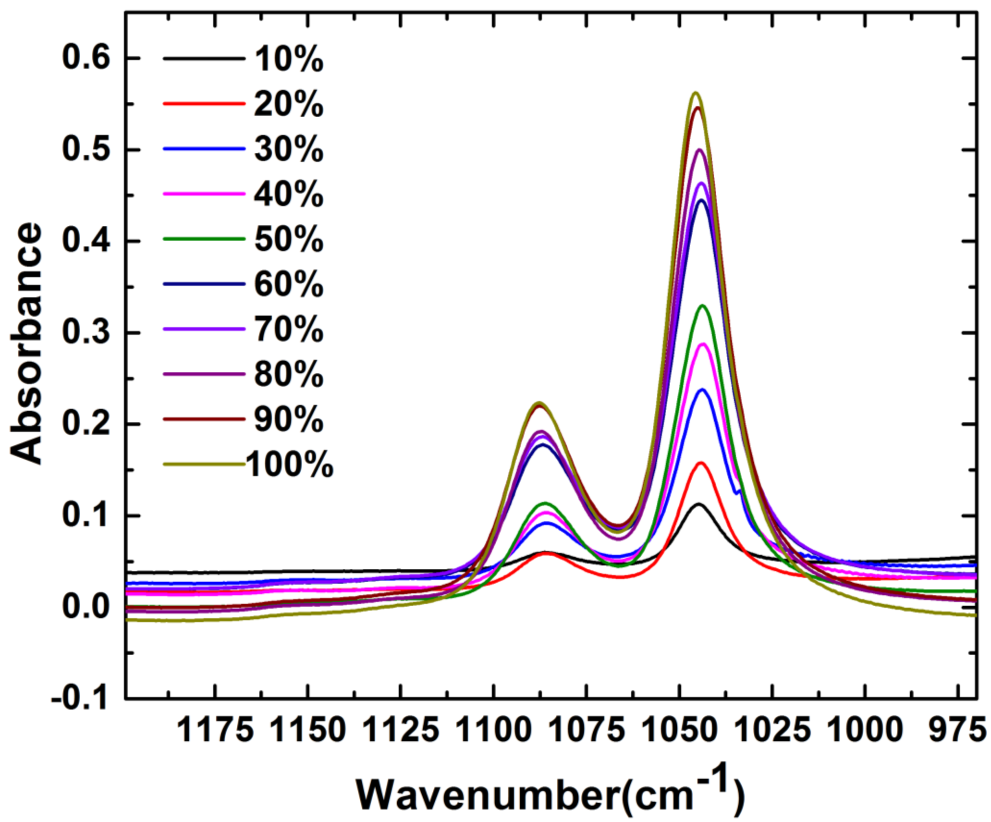

Supplementary Figure S4: ATR-FTIR spectrum of EtOH-water mixture as concentration of EtOH changes from 10 wt% to 100 wt%.

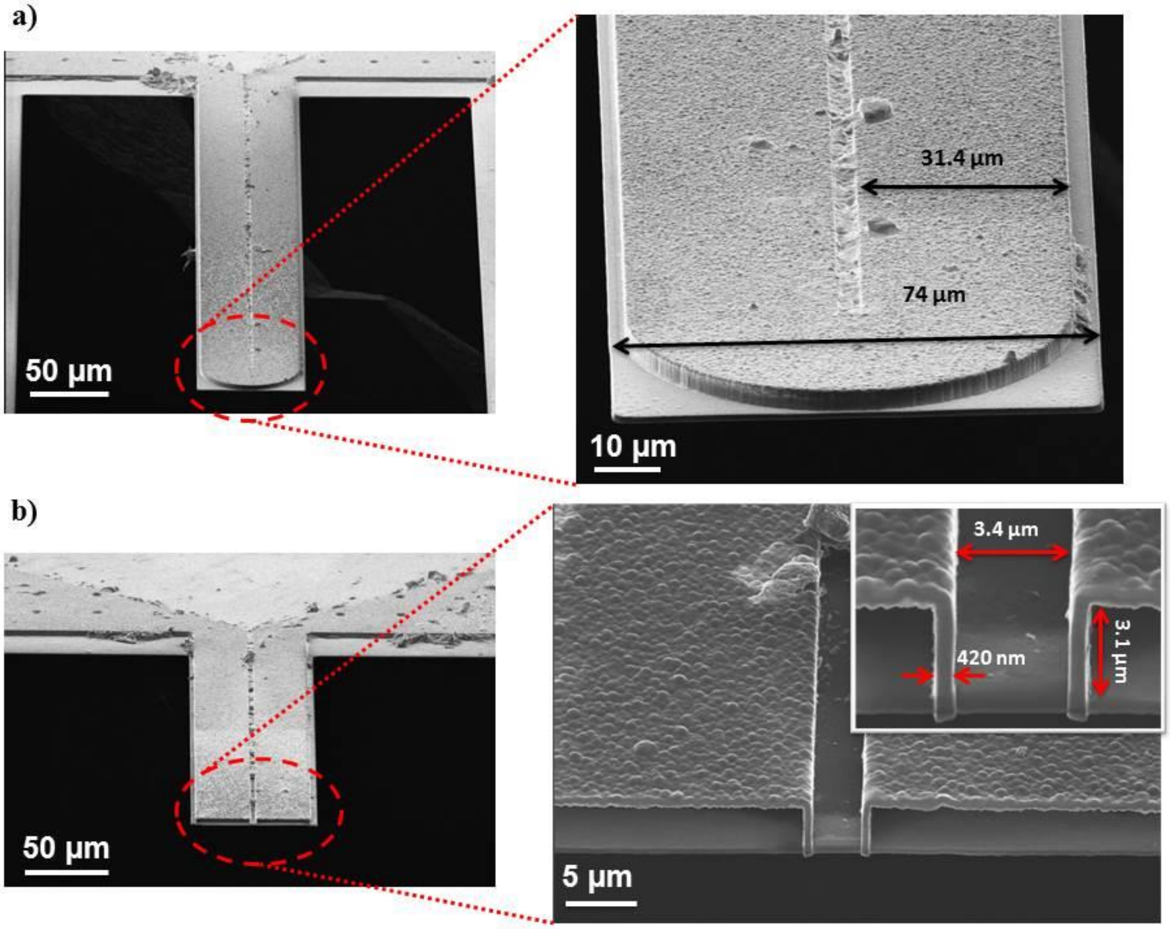

Supplementary Figure S5: Scanning Helium Ion Microscope (HiM) image of a microfluidic cantilever a) top view of the microfluidic cantilever and b) top view of a cut microfluidic cantilever and cross section of the channel constructed on the top of cantilever.

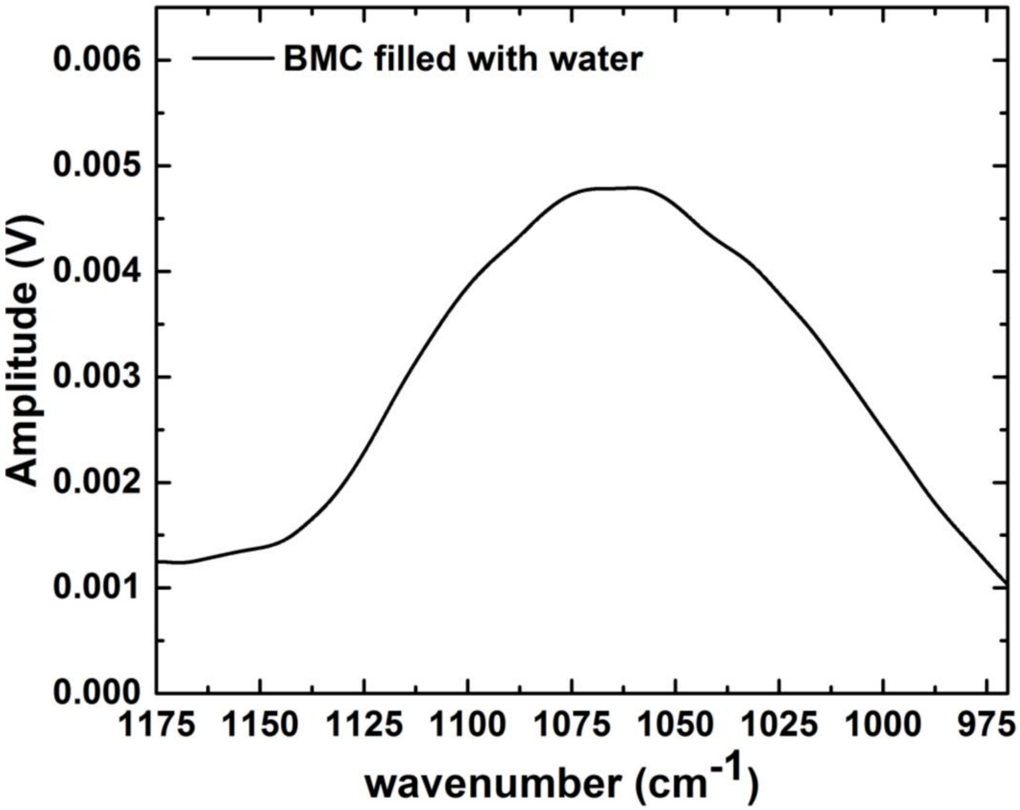

Supplementary Figure S6: PMCDS spectrum of bimaterial microfluidic cantilevers (BMC) filled with water used as subtraction reference
